# MiR-3162-3p Is a Novel MicroRNA That Exacerbates Asthma by Regulating β-Catenin

**DOI:** 10.1371/journal.pone.0149257

**Published:** 2016-03-09

**Authors:** Chao Fang, Weihong Lu, Chengyan Li, Xi Peng, Yang Wang, Xiulan Huang, Zhihong Yao, Nali Cai, Yuge Huang, Xingliang Zhang, Jianxin Tan

**Affiliations:** 1 Department of Pediatrics, Affiliated Hospital of Guangdong Medical College, Zhanjiang, 524001, China; 2 Department of Biomedical Research, National Jewish Health, Denver, Colorado, 80206, United States of America; French National Centre for Scientific Research, FRANCE

## Abstract

Asthma is a common chronic respiratory disease. In a previous study, we found several circulating microRNA signatures associated with childhood asthma and selected miR-3162-3p for subsequent studies. Since the target proteins and underlying molecular mechanisms of miR-3162-3p in asthma etiopathogenesis are not well characterized, we designed this study to clarify its role. We employed bioinformatics and quantitative PCR methods as a first step to determine the target of miR-3162-3p, and we elucidated β-catenin. Luciferase assays and western blot analysis confirmed β-catenin as a direct target of miR-3162-3p as the 3’-untranslated region of β-catenin mRNA possesses a specific miR-3162-3p pairing site. The correlation between the expression levels of miR-3162-3p and β-catenin is confirmed by quantitative PCR and western blot studies in A549, Beas-2B and H1299 cell lines and OVA-induced asthma mouse model. Of note, upregulation of the endogenous miR-3162-3p level is concomitant with the reduction of β-catenin mRNA and protein expression levels. MiR-3162-3p antagomir treatment antagonizes the endogenous miR-3162-3p and effectively rescues the attenuation of endogenous β-catenin in OVA-induced asthmatic mice, which alleviates airway hyperresponsiveness and ameliorates airway inflammation. Collectively, our findings suggest a novel relationship between miR-3162-3p and β-catenin and clarify their mechanistic role in asthma etiopathogenesis.

## Introduction

Asthma is a common chronic respiratory disease, characterized by airway inflammation, exaggerated bronchial airway hyperresponsiveness (AHR), and variable airflow obstruction in response to inhaled antigens [1]. Asthma can arise from various causative agents and its molecular mechanism has yet to be definitively characterized. Despite a complex interplay between immunologic and inflammatory mechanisms [2], increasing evidence pinpoints to a critical role of microRNAs (miRNAs), a class of short non-coding RNAs, in asthma etiopathogenesis [3–5]. miRNAs may inhibit translation or mediate the degradation of mRNAs through partial base pairing at the 3’-untranslated regions (3’-UTRs) of mature target mRNA transcripts [6]. miRNA levels may be altered in patients’ blood at the early stages of numerous diseases [7] and could be detected in different cell-free body fluids such as blood sample [8], urine [9] and saliva [10]. This suggests that miRNAs could potentially serve as non-invasive biomarkers for many diseases. Indeed, recent studies reported that miRNAs found in the serum and bronchoalveolar lavage fluid (BALF) could serve as biomarkers to better distinguish between the endotypes of asthma [11,12].

Although the roles of miRNAs in human diseases have been investigated since approximately thirteen years ago, research on their functions in allergic diseases such as asthma had only begun in recent years [1,13,14]. The miRNA profiles were significantly altered in experimental asthma mouse models. For example, the expression levels of miRNA-181a, miR-155, miR-150, miRNA-221, miR-106a, miRNA-221, miR-146a and miR-146b were increased in OVA-induced mouse model of asthma [15–18]; the miR-126, miR-145 and miR-106a expression levels were increased in house dust mite (HDM)-induced experimental asthma model [19–21]; and miR-21 was up-regulated in lung-specific interleukin (IL)-13-induced asthma model [22]. The use of diverse cell types in the studies of the role of miR-375 [23,24] and Let-7 [25,26] in the regulation of asthma pathogenesis prevented a consistent and clear interpretation of the results. Our recent study of the miRNA molecular profiles of childhood asthma revealed that the expression levels of miR-3162-3p, Let-7, miR-494 and miR-1260 were upregulated [27]. However, their molecular targets and mechanism of etiopathogenesis have yet to be characterized.

β-catenin, a vital component of β-catenin signaling, has wide-ranging implications in health and disease. It controls many physiological and pathological processes including intercellular adhesion, signal transmission, cell cycle regulation, development and differentiation, tumor formation, angiogenesis, apoptosis and necrosis [28,29]. In addition, a wide range of cytokines, growth factors and inflammatory mediators can regulate β-catenin signaling in airway remodeling in asthma [30]. β-catenin signaling is activated in response to TGF-β1 in airway smooth muscle cells, which is crucial for regulating extracellular matrix production [31]. IL-6 significantly enhances cell motility of BEAS-2B cell via the Akt/GSK-3β/β-catenin Signaling [32]. Moreover, several lines of evidence proved the relevance of β-catenin signaling in asthma. For example, both the protein expression and distribution of β-catenin in toluene diisocyanate (TDI)-induced asthma model were altered [33]; WNT/β-catenin pathway was reported to be significantly modulated in asthma patients and LPS-stimulated RAW264.7 macrophage cell line [34]; the polymorphism of β-catenin promoter affected its mRNA expression levels and contributed significantly to the risk of asthma in Korean subjects [35]; β-catenin signaling was activated in *A*.*fumigatus*-induced asthma mouse [36]; an approximate two-fold higher abundance of WNT-5A, a major ligand in the Wnt/β-catenin signaling, in the airway of smooth muscle cells was observed in asthmatic individuals compared to non-asthmatic individuals [37]. Nonetheless, overexpression of WNT, which prevented the degradation of β-catenin during the allergen challenge phase, attenuated the development of airway inflammation in an acute model [38]. Therefore, a clear functional role of β-catenin signaling in experimental asthma has yet to be established.

To date, several miRNAs associated with disease development were found to target β-catenin signaling [39,40]. For example, miR-200a [41,42], miR-34 [43–45], miRNA-1826[46] and miR-29c [47] regulate epithelial-mesenchymal transition in various cancers by directly targeting β-catenin. However, little is known about the impact of miRNA on asthma etiopathogenesis via the regulation of the β-catenin signaling pathway. In this study, we performed cellular studies and luciferase assays to determine the target of miR-3162-3p. Next, we examined the levels of endogenous miR-3162-3p and β-catenin protein in asthma mouse models to determine whether a correlation exists between them. In addition, we treated asthmatic mice with the miR-3162-3p antagomir and then detected the changes of airway hyperresponsiveness, airway inflammation and endogenous β-catenin. Our study suggests that miR-3162-3p has a potentially significant role in regulating asthma through the direct repression of β-catenin expression.

## Materials and Methods

### Animals, Reagents and antibodies

Female BALB/c mice (6~8 weeks old, 20 ± 2 g) were obtained from the Laboratory Animal Centre of Guangdong Medical College. All mice with access to chow and water ad libitum were acclimated to laboratory conditions (50±10% humidity and 14/10 h light/dark cycles at 22±1°C) for at least one week before use in experiments. The mice were euthanized by cervical dislocation after intraperitoneal administration of 1% pentobarbital sodium (50 mg/kg, Sigma, P3761) after the experimental endpoint. Mice did not become severely ill or moribund during the experiments. All procedures involving animals were approved by the Laboratory Animal Centre of Guangdong Medical College Animal Care and Use Committee and conform to the Guide for Care and Use of Laboratory Animals.

Anti-β-catenin polyclonal antibody (cell signaling 8480), anti-β-actin antibody (cell signaling 4967S), HRP-conjugated anti-mouse IgG (CST, 7076), and HRP-conjugated anti-rabbit IgG (CST, 7074) were used for western blot. Kits used included miRNA RT-PCR Kit (Takara, RR716), miRcute miRNA Kit (Tiangen, DP501), and Dual-Luciferase Reporter Assay System (Promega, E1910). Lipofectamine 3000 (Invitrogen, L3000015) was used for transfection experiments. All oligonucleotide including miRNA mimic, miRNA inhibitor and their controls were chemically synthesized by Biomics Biotechnologies Co., Ltd. (Suzhou, China).

### Cell culture and transfection

A549, H1299 and Beas-2B cells were grown in DMEM (Invitrogen) supplemented with 10% fetal calf serum (Hyclone) and 1% penicillin/streptomycin (Beyotime, referred to as complete medium) at 37°C under 5% CO_2_. To assess the transfection efficiency of the miRNA oligonucleotides in the cell lines of interest, we randomly selected mimic-let-7c (a synthetic miRNA hsa-let-7c-3p mimic oligonucleotide), mimic-miR-3162-3p (a synthetic miRNA hsa-miR-3162-3p mimic oligonucleotide) and their syn-miR controls (negative control, NC, consisting of a scrambled oligonucleotide) for transfection into A549 and HEK293 cells (S1 Fig). The transfection efficiency was quantified by quantitative real-time RT-PCR (qRT-PCR). Subsequently, A549, H1299 and Beas-2B cells were seeded (70% confluence) in 24-well plates for the transfection assays. After 24 h, cells were transfected with each chemically synthesized oligonucleotide including mimic-miRs (miRNA mimic), syn-miR control (miRNA mimic control), anti–miRs (miRNA inhibitor), or anti-miR control (miRNA inhibitor control), which were derived from hsa-let-7c, hsa-let-7c-5p, hsa-miR-3162-3p, hsa-miR-1260p and hsa-miR-494 (seen in Supporting Information).

### Plasmids construction and luciferase reporter system assay

The oligonucleotide sequences of the two regions in the 3′-UTR of the human β-catenin mRNA (NM_001904) that can base pair with hsa-miR-3162-3p, flanked by the *NheI* and *SalI* restriction sites, are: 5′-CTAGCGGATCCAACTTCAGAAAGACTTGGTTGGTAGGGTGGGTGGG-3′ (Oligo 1, F); 5′-TCGACCCACCCACCCTACCAACCAAGTCTTTCTGAAGTTGGATCCG-3′ (Oligo 1, R); 5′-CTAGCGGATCCAACTATTTGGGATATGTATGGGTAGGGTAAG-3′ (Oligo 2, F); 5′-TCGACTTACCCTACCCATACATATCCCAAATAGTTGGATCCG-3′ (Oligo 2, R). The oligonucleotide sequences of the corresponding mutant constructs generated by mutating the seed regions of the miR-3162-3p pairing site are: 5′-CTAGCGGATCCAACTTCAGAAAGACTTGGTTCCATCACTGGGTGGG-3′ (Mut-site 1, F); 5′-TCGACCCACCCAGTGATGG AACCAAGTCTTTCTGAAGTTGGATCCG-3′ (Mut-site 1, R); 5′-CTAGCGGATCCAACTATTTGGGATATGTATGCCATCACTAAG-3′ (Mut-site 2, F); 5′-TCGACTTA GTGATGGCATACATATCCCAAATAGTTGGATCCG-3′ (Mut-site 2, R). The above oligonucleotides were chemically synthesized by Sangon (Shanghai, China) and then cloned into the pmirGLO dual-Luciferase miRNA target expression vector (Promega, E1330). All constructs were verified by *BamHI* digestion and direct sequencing.

Cells were seeded in 24-well plates one day before transfection. The cells were then transiently co-transfected with 1.0 μg wild type reporter plasmid or mutant plasmid in the presence of 50 nmol/L syn-miR controls, 50 nmol/L mimic-miR-3162-3p, 100 nmol/L anti-miR control or 100 nmol/L anti-miR-3162-3p using lipofectamine 3000 (Invitrogen). Firefly and Renilla luciferase activities were measured consecutively by the Dual-Luciferase Reporter Assay System (Promega, E1910) according to the manufacturer′s instructions. Three independent experiments were performed.

### Mature miRNAs and Gene Expression Analysis by qRT-PCR

Total RNA was isolated with Trizol (Life Technology), followed by DNase treatment to eliminate contaminating genomic DNA, and then subjected to a reverse transcription reaction. Relative gene expression was determined by two-step qRT-PCR. Quantitative PCRs for miRNA and mRNA were performed with SYBR PrimeScript miRNA RT-PCR Kit (TaKaRa, RR716) and PrimeScript RT reagent Kit with gDNA Eraser(RR047A) on a ROCHE LightCycler480II Real Time PCR System, respectively. The 2^-ΔΔCt^ method was used to analyze the relative changes which were normalized to the RNU6B endogenous control. The qPCR Primers were purchased from Sangon (Shanghai, China) (seen in Supporting Information). Three independent experiments were performed in triplicate.

### Allergic asthma model to ovalbumin (OVA) in mice and treatment of asthmatic mice with miR-3162-3p antagomir

The OVA-induced asthma mouse model was established as previously described [48]. 6 to 8-week-old female BALB/c (20 ± 2 g) mice in the asthmatic group were sensitized by intraperitoneal injection of 50 μg OVA (Sigma, St. Louis, MO, USA) emulsified in 2 mg aluminum hydroxide (Aladdin, Shanghai, China) in 200 μl normal saline on days 0, 7 and 14. Mice in a nebulizer (KYWH1004, Foshan Turning Medical Technology Co., Ltd, Foshan, China) were challenged with 1% OVA (w/v, in PBS) for a 30 min duration on days 21, 22 and 23. For the remission group, the mice were fed for 7 days after OVA sensitization and challenge. Normal saline instead of 1% OVA was used as the challenge solution for the sensitized mice group. In the normal control group, normal saline instead of 1% OVA was used as both sensitization and challenge solutions. The miR-3162-3p antagomir and its control oligonucleotides were chemically synthesized and specially modified for animals transfection by Biomics Biotechnologies Co., Ltd. (Suzhou, China). For both the Asthma + antagomir group and the Asthma + antagomir control group, the antagomir or its control oligonucleotides was dissolved in endotoxin-free water, and the working dilution was prepa red in animals RNA transfection reagent Entranster^™^-in vivo (18668-11-1, Engreen Biosystem Co, Ltd). Each of both oligonucleotides working dilution was intraperitoneally administered to asthmatic mice at a dosage of 4.0 mg/kg on days 20, 21, 22 and 23 (Table 1). Behavioral features of each group of mice during the challenge phase were recorded (S1 Table).

**Table 1:** Treatments for five groups of mice (n = 6–8).

Groups	Sensitization (d0, d7, d14)	Challenge (d21, d22, d23)	Administration (d20 to d23)	Remission (d24 to d30)
**Normal**	—	—	—	—
**Sensitized Control**	^*a*^saline	—	—	—
**Sensitization**	^*a*^OVA	—	—	—
**Asthmatic Control**	^*a*^OVA	^*b*^saline	—	—
**Asthma**	^*a*^OVA	^*b*^OVA	—	—
**Asthma + antagomir**	^*a*^OVA	^*b*^OVA	antagomir	
**Asthma + antagomir control**	^*a*^OVA	^*b*^OVA	antagomir control	
**Remission**	^*a*^OVA	^*b*^OVA	—	normal feeding

^*a*, *b*^: total volume at 0.2 ml and 600 ml, respectively.

### Respiratory responses to methacholine

The procedures for determining methacholine (Mch) responsiveness were described in detail elsewhere [49–51] and our previous publication [48]. Briefly, unrestrained mice were caged in a noninvasive whole-body plethysmograph (Buxco) 24 h after the last treatment, and the enhanced pause value (Penh [52,53]) in the natural state was set as the baseline. Each group of mice inhaled aerosol Mch (0–40 mg/ml Mch) for 2 min, and then the Penh values were recorded for 3 min.

### Histopathology

Lung sections were stained with hematoxylin and eosin (H&E) (Baso, Zhuhai, China) and Periodic Acid-Schiff (PAS) (Baso, Zhuhai, China) solutions to detect eosinophil infiltration and mucosubstances, respectively, as previously described [48].

### Collection of lung, blood, BALF and cell counts

Mice were anesthetized by intraperitoneal injection of 1% pentobarbital sodium (50 mg/kg, Sigma, P3761) 24 h after the last challenge. Blood samples were collected through retro-orbital bleeding. Serum was collected after centrifugation (200 g, 10 min). MiRNAs in serum and lung tissues were extracted using the miRcute miRNA kit. The expression level of miR-3162-3p was examined by qRT-PCR. The lung tissues were collected and stored at −70°C for western blotting. The collection of BALF and cell counts were performed as previously described [48].

### Western blotting

Cells were lysed in RIPA lysis buffer (Beyotime). Equivalent protein quantities (20 μg) were used for the SDS-PAGE, and the electrophoresed samples were transferred to 0.2 μm PVDF membranes (Millipore, Bedford, MA, USA). Membranes were probed with the indicated primary antibodies, followed by appropriate HRP-conjugated secondary antibodies. Immunoreactive bands were visualized with Immobilon Western Chemiluminescent HRP Substrate (Milllipore, WBLUR0500).

### Statistical analysis

The results were analyzed with GraphPad Prism 5.0 and are expressed as the mean ± standard deviation (SD). Correlations between groups were calculated using one-way analysis of variance (ANOVA), followed by Tukey's multiple comparison *post hoc* test. p values < 0.05 were deemed statistically significant.

## Results

### miR-3162-3p represses the mRNA and protein expression of β-catenin

**Fig 1:**
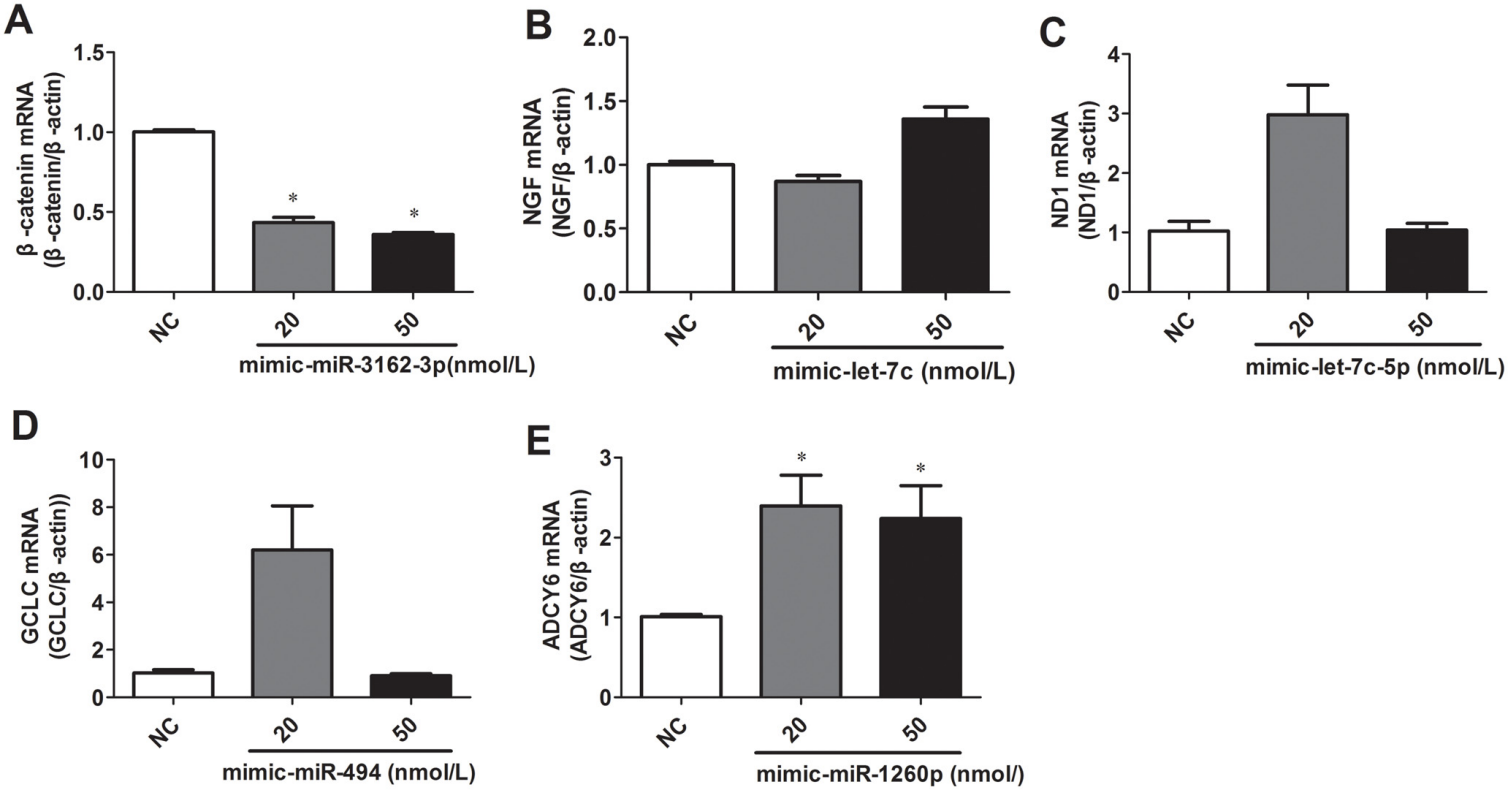
The mRNA expression levels of β-catenin (A), NGF (B), ND1 (C), GCLC (D) and ADCY6 (E) in response to the potentially matched miRNAs mimcs were examined by quantitative real-time RT-PCR. A549 cells were transfected with mimic-miR synthetic oligonucleotides. Syn-miR-control denotes the negative control (NC). The change in the mRNA expression level is expressed as the degree of fold change with respect to NC (2^-ΔΔCt^, mean ± SD, n = 3 independent experiments, *p<0.05 vs. NC). β-actin was used as an internal control.

In our previous study, we compared the miRNA expression profiles between normal and asthma patients by hybridization arrays and quantitative PCR (qPCR). miR-1260a, miR-let-7c-5p, miR-3162-3p and miR-494 were observed to show significantly higher expression levels in asthma patients [27]. To elucidate the underlying link between these upregulated miRNAs and asthma, we screened for potential target genes that contain a highly conserved complementary 3′-UTR sequence (“seed match”) using publicly available bioinformatics tools miRDB [54], PicTar and TargetScreen, as a first step. Next, we searched for potential asthma-related genes in the database for complex diseases: the genetic association database (GAD) [55]. After an exhaustive literature and bioinformatics search, we eventually narrowed down our search to five genes as the potential asthma-related targets and validated them by qPCR. The results showed that β-catenin was downregulated in response to the upregulation of miR-3162-3p (Fig 1). Therefore, we selected miR-3162-3p as the optimal asthma-related candidate for subsequent experiments.

**Fig 2:**
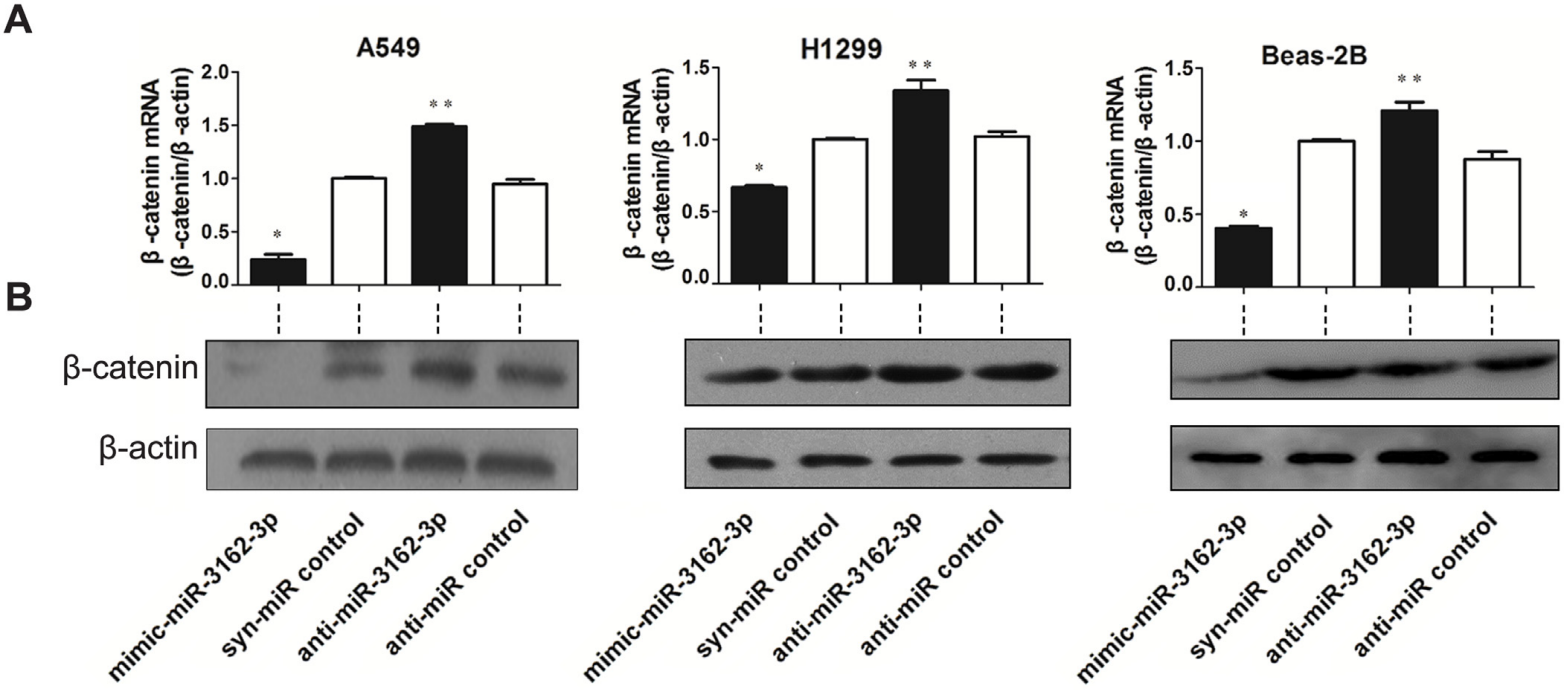
Effects of miR-3162-3p, its mimics and inhibitor on the endogenous β-catenin expression level in A549, H1299 and Beas-2B cell lines. **(A)** Synthetic oligonucleotides—mimic-miR-3162-3p, syn-miR control, anti-miR-3162-3p or anti-miR control—were transfected into A549, H1299 and Beas-2B cell lines, respectively. β-catenin mRNA was quantified by real-time PCR (β-actin as an internal control). Results are shown as the mean ± SD (n = 3), *p<0.05 vs. syn-miR control; **p<0.05 vs. anti-miR control. **(B)** Western blot analysis of cell lysates was performed to determine the relative β-catenin protein expression level.

Next, we studied the effects of miR-3162-3p on β-catenin mRNA expression levels. Each miRNA—mimic-miR-3162-3p (miR-3162-3p mimic), syn-miR-control (NC), anti-miR-3162-3p (miR-3162-3p inhibitor) and anti-miR-control (miR-3162-3p inhibitor control)—was transfected into three pulmonary cell lines A549, H1299 and Beas-2B (Fig 2). Mimic-miR-3162-3p effectively repressed β-catenin mRNA expression compared to the negative control in these three cell lines. anti-miR-3162-3p, a specific endogenous miR-3162-3p inhibitor, was used to evaluate the corresponding change in β-catenin mRNA expression level. β-catenin mRNA expression level was noticeably potentiated in the presence of anti-miR-3162-3p. Comparison with the negative control, anti-miR-control, confirmed that the change in β-catenin mRNA expression level was due to the targeted inhibition of endogenous miR-3162-3p. This suggests that the expression level of endogenous miR-3162-3p is inversely correlated to that of β-catenin mRNA and that the presence of the former causes a reduction in β-catenin mRNA (Fig 2A).

Next, we studied the expression patterns of β-catenin protein in response to miR-3162-3p mimic or inhibitor by western blotting. The addition of mimic-miR-3162-3p resulted in a significant decrease in β-catenin protein expression level in A549, H1299 and Beas-2B cell lines (Fig 2B). Similar to our observations of the reduced β-catenin mRNA expression level, the addition of anti-miR-3162-3p resulted in an increase in β-catenin protein expression level in these three cell lines, as compared to the negative and inhibitor controls (Fig 2B).

### 3’-UTR of β-catenin is the direct target of miR-3162-3p

**Fig 3:**
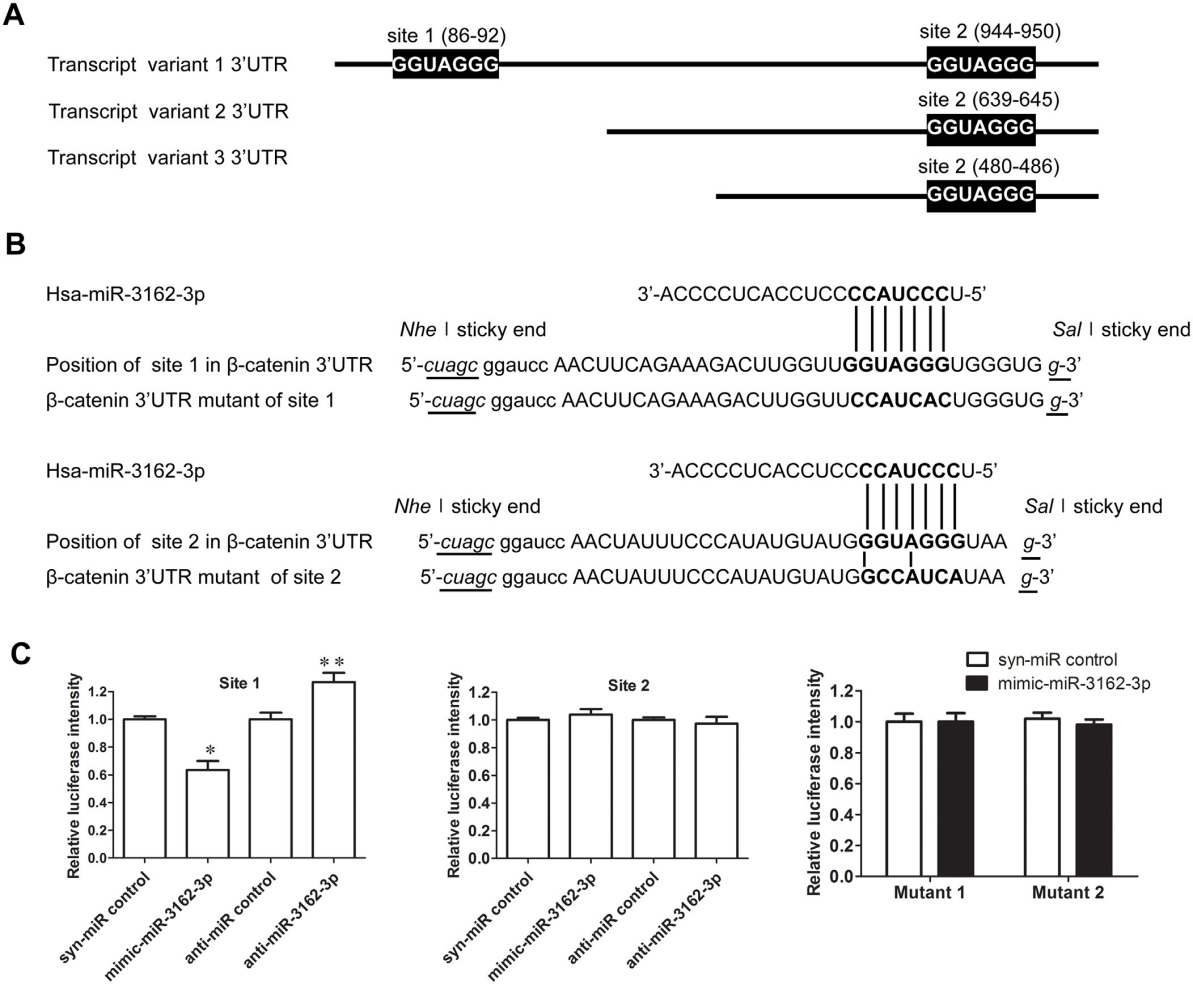
β-catenin is a direct target of miR-3162-3p. **(A)** The three mRNA transcript variants of β-catenin possess different 3’-UTRs, which contain either one or two miR-3162-3p pairing sites. Site 2 is a common pairing site for all three transcript variants. Transcript variants 2 and 3 have only one pairing site (site 2) and transcript variant 1 has two pairing sites, sites 1 and 2. **(B)** Potential matching target sites of miR-3162-3p in the 3’-UTR of β-catenin mRNA transcript and sequence information of the wild type and mutant luciferase report constructs. Two fragments which comprise miR-3162-3p target sites on the 3′-UTR of β-catenin mRNA transcript were separately inserted into the luciferase gene of the pmirGLO vector at the *NheI* and *SalI* restriction sites. **(C)** The physical interaction between miR-3162-3p and the predicted target sites on β-catenin 3′-UTR in A549 cells was detected by luciferase reporter assay. Each wild type reporter construct was co-transfected with mimic-miR-3162-3p, syn-miR control, anti-miR-3162-3p or anti-miR control. The mutant reporter construct was co-transfected with mimic-miR-3162-3p or syn-miR control. Luciferase activity was measured at 24 h after transfection. Activity of the 3’-UTR reporter constructs was normalized to Renilla. Results are shown as the mean ± SD (n = 3). *p < 0.05 vs. syn-miR control.

β-catenin has three transcript variants with different 3'-UTR sequences, but they encode the same protein. Transcript variant 1 contains two miR-3162-3p pairing sites at nucleotides 86–92 (nt86-92) (site 1) and nt945–950 (site 2) within the 3′-UTR. Transcript variants 2 at nt639–645 and 3 at nt480-486 possess the same conserved pairing site as site 2 in transcript variant 1 (Fig 3A).

The predicted interaction between miR-3162-3p and the 3′-UTR of β-catenin was shown in Fig 3B. To clarify the direct target of miR-3162-3p, sites 1 and 2 of the 3′-UTR of β-catenin mRNA transcript were inserted separately into the Dual-Luciferase vector pmirGLO, and the ability of miR-3162-3p to regulate the reporter gene was examined. The addition of mimic-miR-3162-3p elicited a conspicuous response for site 1 of the 3′-UTR of β-catenin, where the reporter gene was inhibited (Fig 3C). The inhibition of reporter gene expression was relieved by the addition of anti-miR-3162-3p which specifically targets endogenous miR-3162-3p (Fig 3C). However, site 2 in 3′-UTR of β-catenin did not display a similar response to miR-3162-3p mimic or inhibitor (Fig 3C). To confirm the specific binding of miR-3162-3p to site 1 of the 3′-UTR the β-catenin transcript, several luciferase vectors with mutations in the seed match region were constructed (Fig 3B). Disruption of site 1 affected the recognition of the luciferase reporter by the mimic-miR-3162-3p, whereas substitution of several nucleotides in site 2 did not alter luciferase activity of the reporter gene (Fig 3C). These results indicate that the regulation of luciferase activity depends on the recognition of miR-3162-3p for the conserved pairing site 1 within the 3′-UTR of β-catenin mRNA transcript.

### The endogenous miR-3162-3p level is upregulated whereas the mRNA and protein expression levels of β-catenin are reduced in asthma mouse model

Bioinformatics analysis revealed that the 3’-UTR of both mouse and human β-catenin share common miR-3162-3p binding sites. The asthma mouse model and endogenous miR-3162-3p were characterized in our previous study [48].

**Fig 4:**
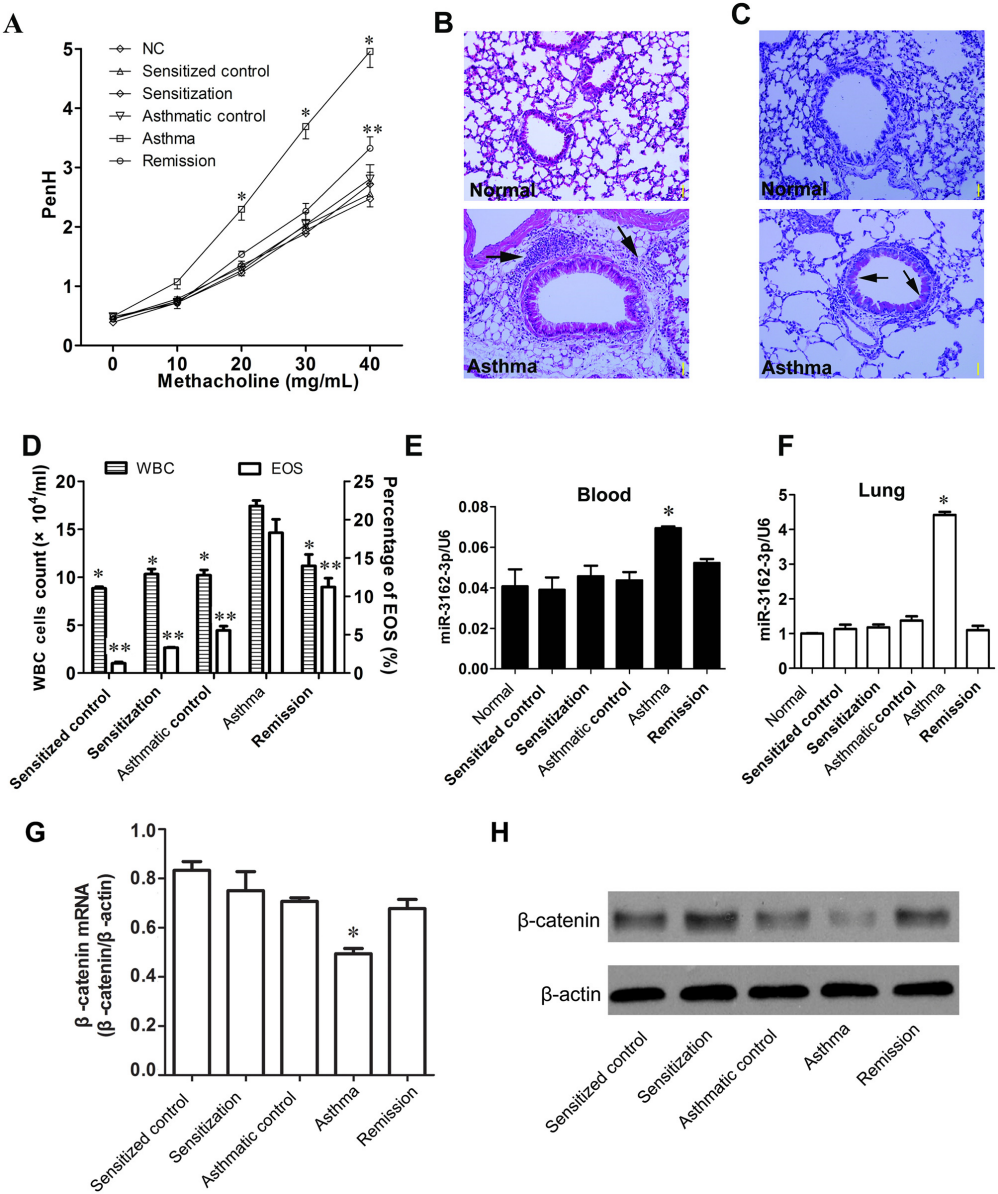
The level of endogenous miR-3162-3p, β-catenin mRNA and protein expressions in the lung in mice varies at different stages of asthma pathogenesis. **(A)** Responsiveness to aerosolized methacholine was assessed by PenH value one day after the last process in each group of mice. The PenH values indicate airway hyperresponsiveness when mice inhaled the aerosolized methacholine. Data is shown as the mean ± SEM. *p < 0.05 vs. other groups; **p<0.05 vs. normal or sensitization (n = 5–6 per group). **(B)** Representative lung sections were H&E-stained to estimate the degree of inflammation (blue arrows) in the terminal bronchioles and alveolar regions of each group of mice (200× magnification). **(C)** Representative airway tissues were stained with PAS to assess the relative amount of intraepithelial mucus production (black arrows). Abundant mucus hypersecretion was found in asthma mice (200× magnification). **(D)** Number of BALF total cells and proportion of eosinophils (EOS) in BALF mice. *p, **p<0.05 vs. challenge. **(E-F)** Expression level of miR-3162-3p in peripheral blood and lung from each group of mice. Results from three independent experiments (n = 3) are shown. *p<0.05 vs. other groups of mice. **(G)** β-catenin mRNA expression profile in the lung for each group is shown. *p < 0.05 vs other groups. **(H)** β-catenin protein level in the lung of asthma mice showed a marked decrease in response to allergen challenge which resulted in upregulation of endogenous miR-3162-3p level.

Behaviors of each group of mice during the challenge phase were recorded (S1 Table): the sensitized control, sensitization and asthmatic control groups displayed relatively normal activities. The asthmatic and the remission groups during the challenge phase showed common characteristics of asthma, such as fidgeting and murmuring, wheezing, silence or sluggish movement, and grasping of the nose and ears. The remission phase effectively restored the asthma mice back to normal. In addition, Mch responsiveness of each group of mice was assessed by a non-invasive method (PenH). One day after the last process, the asthmatic group exhibited an overt response to aerosolized Mch unlike the other groups (Table 1 and Fig 4A). There was substantial inflammatory cell influx in the peribronchiolar and perivascular regions and airspaces in the asthmatic group (Fig 4B). Mucus overproduction, a key characteristic of asthma, was observed in the asthmatic group (Fig 4C). The white blood cell (WBC) and eosinophil (EOS) levels in BALF of each group were counted. The asthmatic group showed a significant increase in the WBC count compared to the other groups, where no obvious difference in WBC count was observed (Fig 4D). The percentages of eosinophils for the sensitization and challenge groups were larger than their corresponding controls. The EOS count in the remission group was relatively lower than that in the asthmatic group, but was much higher than the other groups. Similar with the observation of the WBC count, the EOS level in the challenge group was significantly enhanced as compared to the other groups (Fig 4D). These results convincingly showed that the OVA-mouse model of asthma was successfully established.

To characterize the change in the endogenous miR-3162-3p level in the mouse lung, the levels of miR-3162-3p in serum and lung at different stages of asthma were examined by qPCR. The concentration of miR-3162-3p in the peripheral blood was markedly lower than that in the lung. The asthmatic group displayed the highest level of miR-3162-3p *in vivo* in the peripheral blood and the lung among all groups (Fig 4E and 4F).

To determine the relationship between endogenous miR-3162-3p and β-catenin *in vivo*, we examined the mRNA expression level of β-catenin in each group by qRT-PCR. There was a marked decrease in the β-catenin mRNA expression level in the asthmatic group compared to the other groups (Fig 4G). A distinct opposite trend was observed when the mRNA expression level of β-catenin in the lung and the endogenous miR-3162-3p in the blood and lung at the different stages of asthma were compared (S2 Fig). Next, western blot analysis showed that β-catenin protein expression level exhibited a similar trend as its mRNA expression level, where there was a significant decrease in the β-catenin protein expression level in the asthmatic group compared to the other groups (Fig 4H and S2 Fig).

### MiR-3162-3p antagomir alleviates airway hyperresponsiveness, ameliorates airway inflammation and effectively rescues the attenuation of endogenous β-catenin in OVA-induced asthmatic mice

**Fig 5:**
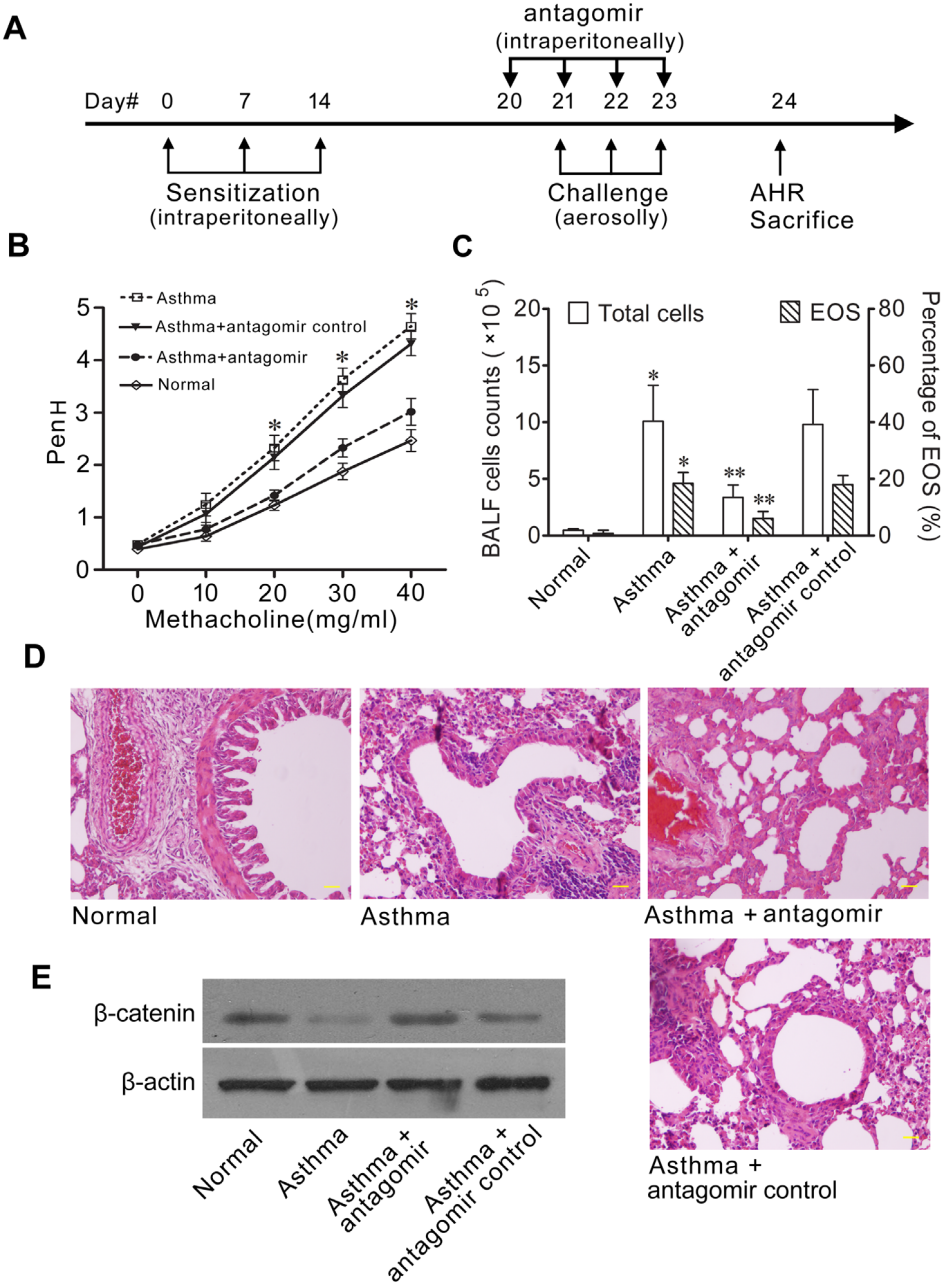
Administration of anti-miR-3162-3p reduces airway hyperresponsiveness and inflammation, whereas rescues the attenuation of endogenous β-catenin expression level in OVA-induced asthma mice. **(A)** Schematic representation of anti-miR-3162-3p delivery in OVA-induced asthmatic mice. **(B)** Anti-miR-3162-3p reduced airway hyperresponsiveness, which was estimated with increasing concentrations of methacholine. **(C)** Anti-miR-3162-3p decreased the total cell and eosinophil numbers (EOS) in BALF in comparison to both the asthmatic mice and the anti-miR control treated asthmatic mice. *p<0.05 vs. Asthma; **p<0.05 vs. Asthma or Asthma + anti-miR control groups. **(D)** Anti-miR-3162-3p suppressed airway inflammatory infiltration from lung of asthmatic mice. Lung sections from different groups stained using hematoxylin and eosin (H&E) were shown (200× magnification). **(E)** Anti-miR-3162-3p counteracted the decrease of endogenous β-catenin protein expression in OVA-induced asthmatic mice lung.

To further validate that the severity of asthma may be attributed to the downregulated β-catenin due to the upregulated endogenous miR-3162-3p in OVA-induced asthmatic mice, the antagomir of miR-3162-3p was administrated to asthma mice (Fig 5A), and then examined airway hyperresponsiveness (AHR), airway inflammation and β-catenin protein expression level. First of all, to determine the effect of miR-3162-3p antagomir on AHR, Mch responsiveness of four groups of mice was assessed by PenH. The asthma group of mice showed an obvious dose-dependent increase in airway resistance in response to aerosolized Mch compared to the normal group. This obvious increase was mostly reversed near to the normal group by miR-3162-3p antagomir treatment but not antagomir control (Fig 5B and Table 1). Moreover, to evaluate the role of miR-3162-3p in airway inflammation, BALF total cells and EOS cells were counted, and then H&E staining of the lung sections was performed. BALF total cells and EOS cells were more in the asthmatic group than the normal control. MiR-3162-3p antagomir administration decreased the numbers of BALF total cells and EOS cells in the asthmatic group in comparison to the antagomir control (Fig 5C). The inflammatory infiltration in both perivascular and peribronchial regions in the asthmatic group was significantly decelerated by miR-3162-3p antagomir delivery but not its control oligonucleotide (Fig 5D). Finally, the protein expression level of β-catenin in lung was detected by western blot. The β-catenin protein expression was downregulated in OVA-induced asthmatic mice. MiR-3162-3p antagomir treatment relieved the downregulation of β-catenin expression in asthmatic mice (Fig 5E). These data confirmed that the endogenous miR-3162-3p could aggravate the severity of allergic asthma, which could be antagonized by the intervention of miR-3162-3p antagomir.

## Discussion

Abnormal expression of several miRNAs has been detected in the airways, BALF or lymphocytes of asthma patients and in different asthma mouse models [1,56]. So far, around ten miRNAs have been reported to play a role in asthma pathogenesis and the vast majority of asthma-related miRNAs were validated in HDM- or OVA-induced asthma mouse models [1,16,21]. Of note, we used hybridization arrays to compare the miRNA expression profiles between juvenile asthma patients and their counterparts in our previous study, and found that the levels of miR-3162-3p could be used to differentiate childhood asthma patients from healthy subjects [27]. To our knowledge, miR-3162-3p was implicated in multidrug resistance in gastric carcinoma [57]. In this study, we identified an additional novel role for miR-3162-3p in asthma etiopathogenesis. Only five proteins have been shown to be the direct targets of the known asthma-related miRNA [1]. Here, we confirmed that the highly conserved sequence, site 1, within the 3’-UTR of the β-catenin transcript is a genuine miR-3162-3p binding site. Furthermore, through the use of miR-3162-3p mimic and inhibitor, we confirmed the relationship between β-catenin and miR-3162-3p in A549, Beas-2B and H1299 cell lines. The increased β-catenin expression was observed in *A*.*fumigatus*-induced asthma mouse [36], whereas we found that β-catenin expression was decreased in OVA-indcued asthma mouse model. In addition, we demonstrated that the endogenous miR-3162-3p could aggravate the severity of allergic asthma by miR-3162-3p antagomir relieving the reduction of β-catenin expression in asthmatic mice and suppressing airway hyperresponsiveness and airway inflammation. In good accordance with our study, stabilizing β-catenin in the allergen challenge phase attenuated the development of airway inflammation in an acute asthma model [38]. Thus, β-catenin signaling appears to be central in different allergen-induced asthma models. The endogenous miR-3162-3p level in the remission group was observed to decrease significantly seven days after the challenge phase, indicating that sustained changes in miRNA may not be essential for asthma pathogenesis. This is consistent with the current opinion of the field about chronic asthma [56]. Collectively, our study established a relationship between miR-3162-3p and β-catenin signaling in asthma etiopathogenesis.

β-catenin was reported to modulate the remodeling of asthmatic airways [30] and play a role in inflammatory responses, wherein it can prevent the overproduction of IL-6 and NF-κB in LPS-induced inflammatory responses [34]. Remodeling of asthmatic airways and inflammation are involved in asthma pathogenesis and both can occur independently [58]. Therefore, future research could focus on how miR-3162-3p targets the β-catenin signaling pathway to regulate asthma pathogenesis. Since a particular miRNA can target hundreds of genes [14], research can be directed to the elucidation and investigation of the potential target proteins of miR-3162-3p. Also, the use of miRNAs shows great promise in disease diagnosis and therapy as they have been shown to cause minimal side effects [1]. Some small inhibitors that target β-catenin directly or indirectly were reported to be effective in suppressing fibroproliferation of cancer and fibrosis in animal models, but most of which have not been assessed in asthma models yet [30,59]. Thus, specific miRNAs and small inhibitors targeting miR-3162-3p and β-catenin or its cofactors, respectively, are potential areas for future studies

## Supporting Information

S1 Fig Screening for the optimal concentration of synthetic oligonucleotides required for transfection efficiency. Mimic-let-7c-3p, mimic-miR-3162-3p, syn-miR control (negative control, NC), and BLANK (without synthetic oligonucleotides) were individually transfected into A549 or HEK293 cells. *(A)* 50 nmol/L of mimic-let-7c showed satisfactory transfection efficiency with relatively less adverse effects on cell viability compared to 100 nmol/L. *p < 0.05 vs. BLANK or NC; **p < 0.05 vs 20 nmol/L or 50 nmol/L. *(B-C)* Transfection efficiency of 20 nmol/L or 50 nmol/L of mimic-miR-3162-3p transfected into A549 cells and HEK293 cells is shown. *p < 0.05 vs. BLANK or NC. Results are shown as the mean ± SD (n = 3).
(DOCX)

S2 Fig Increase in miR-3162-3p level reduces endogenous β-catenin expression level in asthma mice. **(A)** The endogenous miR-3162-3p level in both blood and lung was upregulated in response to allergen challenge. This is inversely correlated with the β-catenin mRNA expression level in the lung, which was downregulated. *p, **p, ***p<0.05 vs other groups. **(B)** β-catenin protein in lung of asthma mice was markedly increased compared with other groups of mice.
(DOCX)

S1 Table Behavioral features of each group of mice during the challenge phase. (DOCX)

S1 Text Detailed information about Primers for qRT-PCR and miRNA mimic and inhibitor oligonucleotide sequences. (DOCX)
