# Correction: MiR-3162-3p Is a Novel MicroRNA That Exacerbates Asthma by Regulating β-Catenin

**DOI:** 10.1371/journal.pone.0153734

**Published:** 2016-04-11

**Authors:** The PLOS ONE Staff

The last author, Jianxin Tan, should also be listed as a corresponding author. His email is jianxin_tan@163.com. The publisher apologizes for the error.
