# Supplementary material for: MiR-3162-3p Is a Novel MicroRNA That Exacerbates Asthma by Regulating β-Catenin
Source: PLoS One. 2016 Mar 9;11(3):e0149257. doi: 10.1371/journal.pone.0149257 (PMC4784915; doi:10.1371/journal.pone.0149257)
Supplement: S1 Fig — Mimic-let-7c-3p, mimic-miR-3162-3p, syn-miR control (negative control, NC), and BLANK (without synthetic oligonucleotides) were individually transfected into A549 or HEK293 cells. (A) 50 nmol/L of mimic-let-7c showed satisfactory transfection efficiency with relatively less adverse effects on cell viability compared to 100 nmol/L. *p < 0.05 vs. BLANK or NC; **p < 0.05 vs 20 nmol/L or 50 nmol/L. (B-C) Transfection efficiency of 20 nmol/L or 50 nmol/L of mimic-miR-3162-3p transfected into A549 cells and HEK293 cells is shown. *p < 0.05 vs. BLANK or NC. Results are shown as the mean ± SD (n = 3). (DOCX) [file pone.0149257.s001.docx]

**Fig S1. Screening for the optimal concentration of synthetic oligonucleotides required for transfection efficiency**. Mimic-let-7c-3p, mimic-miR-3162-3p, syn-miR control (negative control, NC), and BLANK (without synthetic oligonucleotides) were individually transfected into A549 or HEK293 cells. *(A)* 50 nmol/L of mimic-let-7c showed satisfactory transfection efficiency with relatively less adverse effects on cell viability compared to 100 nmol/L. *p < 0.05 vs. BLANK or NC; **p < 0.05 vs 20 nmol/L or 50 nmol/L. *(B-C)* Transfection efficiency of 20 nmol/L or 50 nmol/L of mimic-miR-3162-3p transfected into A549 cells and HEK293 cells is shown. *p < 0.05 vs. BLANK or NC. Results are shown as the mean ± SD (n = 3).

**
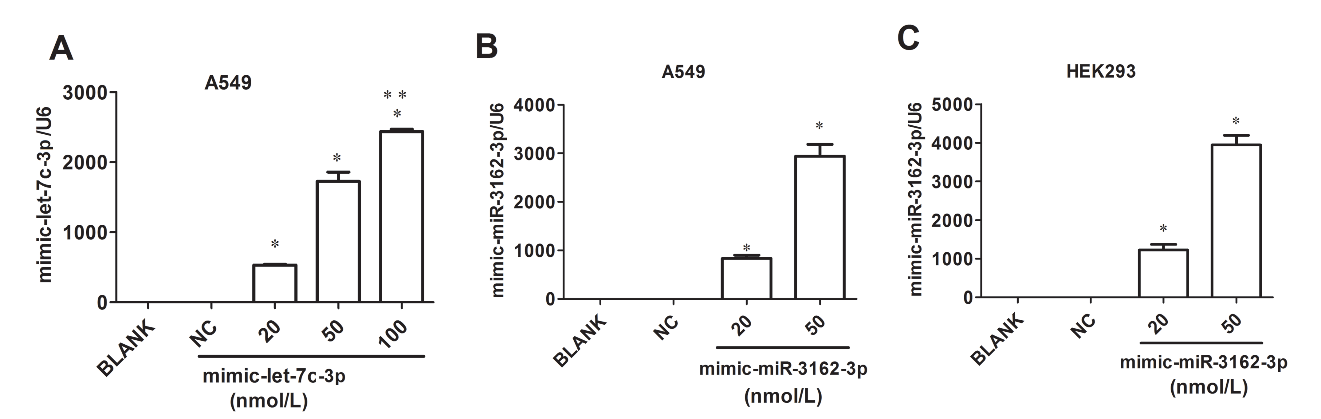
**
