# Supplementary material for: MiR-3162-3p Is a Novel MicroRNA That Exacerbates Asthma by Regulating β-Catenin
Source: PLoS One. 2016 Mar 9;11(3):e0149257. doi: 10.1371/journal.pone.0149257 (PMC4784915; doi:10.1371/journal.pone.0149257)
Supplement: S2 Fig — (A) The endogenous miR-3162-3p level in both blood and lung was upregulated in response to allergen challenge. This is inversely correlated with the β-catenin mRNA expression level in the lung, which was downregulated. *p, **p, ***p<0.05 vs other groups. (B) β-catenin protein in lung of asthma mice was markedly increased compared with other groups of mice. (DOCX) [file pone.0149257.s002.docx]

**Fig S2. Increase in miR-3162-3p level reduces endogenous β-catenin expression level in asthma mice. (A)** The endogenous miR-3162-3p level in both blood and lung was upregulated in response to allergen challenge. This is inversely correlated with the β-catenin mRNA expression level in the lung, which was downregulated. *p, **p, ***p＜0.05 vs other groups. **(B)** β-catenin protein in lung of asthma mice was markedly increased compared with other groups of mice.

**
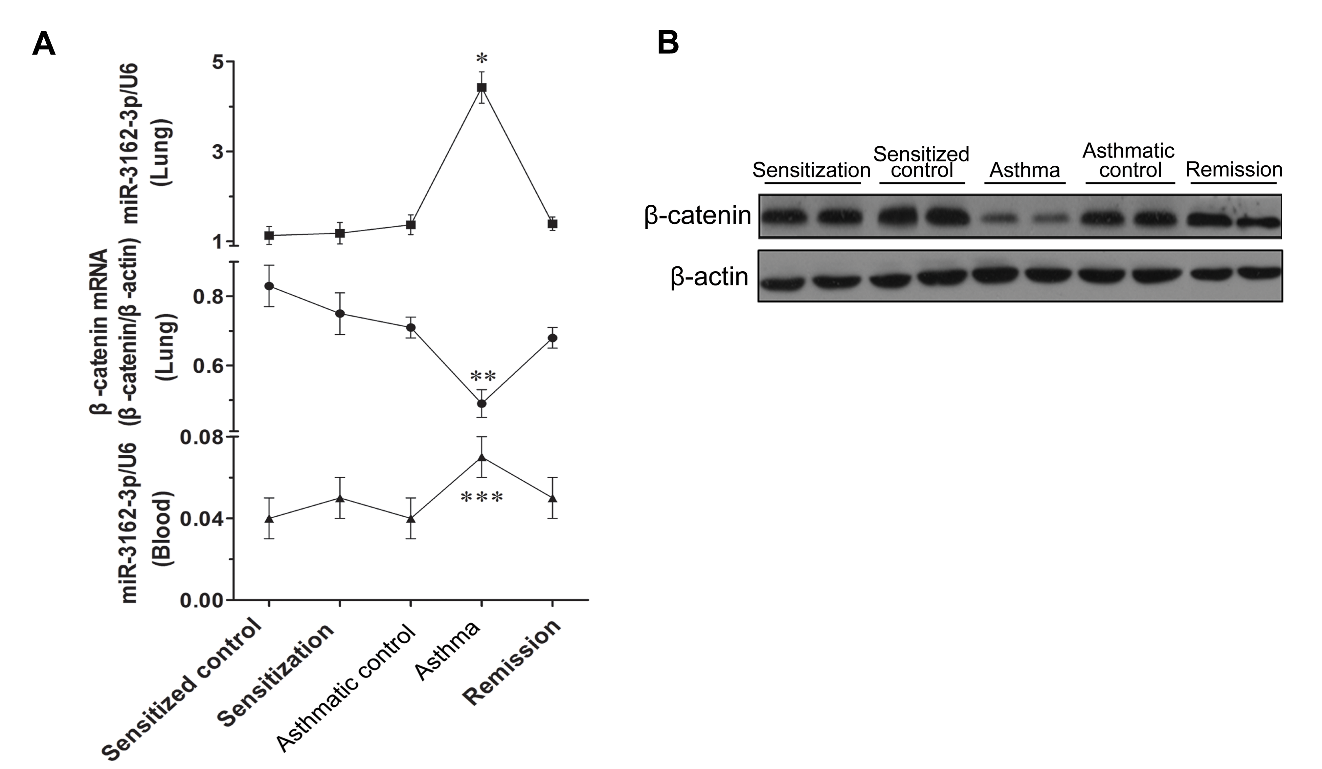
**
