# Supplementary material for: MiR-3162-3p Is a Novel MicroRNA That Exacerbates Asthma by Regulating β-Catenin
Source: PLoS One. 2016 Mar 9;11(3):e0149257. doi: 10.1371/journal.pone.0149257 (PMC4784915; doi:10.1371/journal.pone.0149257)
Supplement: S1 Table — (DOCX) [file pone.0149257.s003.docx]

**Table S1.** Behavioral features of each group of mice during the challenge phase.

| **Groups** | **Challenge** (d21, d22, d23) | **Remission** (d24 to d30) |
| --- | --- | --- |
| **Normal** | normal | — |
| **Sensitized Control** | + | — |
| **Sensitization** | + | — |
| **Asthmatic Control** | + | — |
| **Asthma** | +, +, +, +, + | — |
| **Remission** | +, +, +, +, + | nearly normal |

Five [plus](javascript:void(0);) [sign](javascript:void(0);)s (+, +, +, +, +) denote that behaviors such as wheezing, grasping of nose and ears, fidgeting, murmuring, sluggish movement often happened, while one plus sign (+) denotes that some of the above behaviors occasionally appeared. Behavioral features of each group were observed and recorded at 8:00-9:00 am and 8:00-9:00 pm during challenge phase.
