# Supplementary material for: MiR-3162-3p Is a Novel MicroRNA That Exacerbates Asthma by Regulating β-Catenin
Source: PLoS One. 2016 Mar 9;11(3):e0149257. doi: 10.1371/journal.pone.0149257 (PMC4784915; doi:10.1371/journal.pone.0149257)
Supplement: S1 Text — (DOCX) [file pone.0149257.s004.docx]

**Primers for qRT-PCR and miRNA mimic and inhibitor oligonucleotide sequences**

The qRT-PCR Primers include miR-3162-3p: F 5’-UCCCUACCCCUCCACUCCCCA-3’, R 5’-CTACCCCTCCACTCCCCAAAA-3’; hsa-miR-1260a: F 5’-AUCCCACCUCUGCCACCA-3’, R 5’-GTTGACCTCTGCCACCAAAA-3’; hsa-miR-494: F 5’-UGAAACAUACACGGGAAACCUC-3’, R 5’-TGAAACATACACGGGAAACCTC-3’; hsa-let-7c-5p: F 5’-UGAGGUAGUAGGUUGUAUGGUU -3’, R 5’-TCAGGTAGTAGGTTGTATGGTT-3’; hsa-let-7c-3p: F 5’-CUGUACAACCUUCUAGCUUUCC-3’, R 5’-GTCGTACCTTCTAGCTTTCC-3’; RNU6B: F 5’-CTCGCTTCGGCAGCACA-3’, R 5’-AACGCTTCACGAATTTGCGT-3’; β-catenin: F 5’-TGGTGACAGGGAAGACATCA-3’, R 5’-CCATAGTGAAGGCGAACTGC-3’; GCLC: F 5’-ACCATCATCAATGGGAAGGA-3’, R 5’-TCATCCATCTGGCAACTGTC-3’; NGF: F 5’-GGTGCATAGCGTAATGTCCA-3’, R 5’-CAGTGTCAAGGGAATGCTGA-3’; ADCY6: F 5’-TGGCTTCTTCCAATGAGACC-3’, R 5’-CCCTGTTGCCTGTAGTTTCC-3’; ND1: F 5’-CAAACTCAAACTACGCCCTGA-3’, R 5’-AGAGGTGTTCTTGTGCTGTGA-3’; β-actin: F 5’-TCCGTAAAGACCTCTATGCCAACA-3’, R 5’- GCTAGGAGCCAGAGCAGTAATCTC-3’.

All oligonucleotide sequences are specified as follow: mimic-miR-3162-3p (has-miR-3162-3p mimic): F 5’-UCCCUACCCCUCCACUCCCCA-3’, R 5’-UGGGGAGUGGAGGGGUAGGGA-3’; anti-miR-3162-3p (has-miR-3162-3p inhibitor): 5’-UGGGGAGUGGAGGGGUAGGGA-3’; mimic-let-7c-3p (hsa-let-7c-3p mimic): F 5’-CUGUACAACCUUCUAGCUUUCC-3’, R 5’-GGAAAGCUAGAAGGUUGUACAG-3’; mimic-let-7c-5p (hsa-let-7c-5p mimic): F 5’-UGAGGUAGUAGGUUGUAUGGUU-3’, R 5’-AACCAUACAACCUACUACCUCA-3’; mimic-miR-494 (hsa-miR-494 mimic): F 5’-UGAAACAUACACGGGAAACCUC-3’, R 5’-GAGGUUUCCCGUGUAUGUUUCA-3’; mimic-miR-1260 (hsa-miR-1260 mimic): F 5’-AUCCCACCUCUGCCACCA-3’, R 5’-UGGUGGCAGAGGUGGGAU-3’; syn-miR control (miRNA mimic control, negative control, consisting of a scrambled oligonucleotide): F 5’-UCACAACCUCCUAGAAAGAGUAGA-3’, R 5’-UCUACUCUUUCUAGGAGGUUGUGA-3’; anti-miR control (miRNA inhibitor control): 5’-UCUACUCUUUCUAGGAGGUUGUGA-3’.
